# A New Approach to UV Protection by Direct Surface Functionalization of TiO_2_ with the Antioxidant Polyphenol Dihydroxyphenyl Benzimidazole Carboxylic Acid

**DOI:** 10.3390/nano10020231

**Published:** 2020-01-28

**Authors:** Mattia Battistin, Valeria Dissette, Alessandro Bonetto, Elisa Durini, Stefano Manfredini, Antonio Marcomini, Elisa Casagrande, Andrea Brunetta, Paola Ziosi, Sonia Molesini, Riccardo Gavioli, Francesco Nicoli, Silvia Vertuani, Anna Baldisserotto

**Affiliations:** 1 Department of Life Sciences and Biotechnology, Master Course in Cosmetic Science and Technology (COSMAST), University of Ferrara, Via L. Borsari 46, 44121 Ferrara, Italy; mattiabattistin@kalis.it (M.B.); dssvlr@unife.it (V.D.); dre@unife.it (E.D.);; 2 Kalis S.r.l, Via Caodevilla 38, 31040, Onigo di Pederobba (TV), Italy; andrea.brunetta@kalis.it; 3 Department of Environmental Sciences, Informatic and Statistics, University Ca’ Foscari Venice, Vegapark, Via delle Industrie 21/8, 30175 Marghera, Venice, Italy; alessandro.bonetto@unive.it (A.B.); antonio.marcomini@unive.it (A.M.); 4 Ambrosialab Srl, Via Mortara 171, 44121 Ferrara, Italy; paola.ziosi@ambrosialab.it (P.Z.); sonia.molesini@ambrosialab.it (S.M.); 5 DMSN—Department of Molecular Sciences and Nanosystems, University Ca’ Foscari of Venice, Via Torino 155/b, 30172 Venice, Mestre, Italy; 6 Department of Chemical and Pharmaceutical Science, University of Ferrara, Via Fossato di Mortara 64/B, 44121 Ferrara, Italy; gvr@unife.it (R.G.); nclfnc1@unife.it (F.N.)

**Keywords:** TiO_2_, sunscreen, Oxisol, antioxidant, molecular combination, SPF booster, safety

## Abstract

Skin cancer is the most common malignant cancer with an incidence of 1 million cases/year. It is well known that exposure to UV radiation from sunlight leads the most frequent risk factors for several skin disorders including skin cancer. Sunscreen filters represent a valid protection against dangerous effects derived from UV radiation, and they can be divided in organic and inorganic UV filters. Adding, at the product formulation, molecules with booster effect, or also substances that can increase the protecting effectiveness via synergic mechanisms, can further enhance their protection activity. Moreover, this approach leads to develop formulations with high SPF (Sun Protection Factor) with a reduced content of UV filters, this is in line with the recent decisions of yet a few countries (Palau, Thailand, Philippines, and Hawaii) to ban some sunscreen filters to preserve marine environments (i.e., reef). In this work, a new class of sunscreen UV filters has been synthesized, by means the combination of physical filter and Oxisol, an antioxidant molecule with booster effect. In this study, the synthesis of new physical multifunctional ingredients is reported, by means the direct surface functionalization of inorganic filters (in particular TiO_2_) with Oxisol. In this study, the full characterization of these multifunctional ingredients is also reported, in addition to the cytotoxicity tests, the photocatalytic activity and the rheological properties involved on skin application.

## 1. Introduction

The electromagnetic radiation given off by the Sun can be divided in three main classes: infrared (IR), visible (Vis), and ultraviolet light (UV). It is well known that UV radiation represents the most frequent risk factor for several skin disorder because responsible both mutagen and non-specific damaging effects on epidermal tissues. Part of this UV radiation is absorbed by oxygen molecules in the stratosphere, during the ozone-oxygen cycle (UVC, 100–290 nm) and by ozone (UVB 290–320 nm). The remaining UVA radiation (UVA-II, 320–340 nm, UVA-I, 340–400 nm) and a minimal part of UVB reach the skin and cause metabolic and biological reactions [1]. 

The short-term effects of radiation on the skin may be attributable to UVB radiation; these include the positive one such as the synthesis of cholecalciferol (vitamin D) and, whereas at high doses of UVB, the negative one: the possibility of developing erythema. The long-term effects of high doses of solar radiation are very dangerous and include different skin degenerative changes, including skin photo-aging, actinic keratosis, and development of cancer. Moreover, skin elasticity can also be altered upon exposure to UVA and UVB radiation, due to the production of reactive oxygen species (ROS) that damage collagen and other protein matrices [2,3].

Sunscreen products are widely used against the harmful effects of UV radiation, providing an effective protection towards UVA and UVB. The UV filters, commonly employed in the sunscreen products, can be divided in two main categories.

The classification in inorganic or organic chemical filters is the standard subdivision, according to their physicochemical properties. They are constituents present in topical formulations that have the capacity to interact with UVR via three fundamental mechanisms: reflection, scattering, and absorption.

As an example of inorganic filters, in ZnO and TiO_2_ reflection/scattering, and absorption largely depends on the size: as little as more is the absorption mechanism. Curtis C. et al. have nicely demonstrated this in a recent study [4]. 

Moreover, Sayre and Kollias showed that these properties of inorganic filters maybe found into absorptive semiconductor materials including TiO_2_ and ZnO and non-absorptive scattering materials such as barium sulfate and talc [5]. 

Finally, hydroxyapatite (Ca_10_(PO4)_6_(OH)_2_, HAp) is a calcium phosphate with high biocompatibility, is another good example of the two sub-categories: it can be used as itself or modified, as a booster in sunscreens for its reflecting capabilities. Only when doped with iron or zinc it became also an absorber [6]. 

Scattering phenomena happen when the electric dipole of the particles oscillates with the same frequency of the incident radiation. The result of this interaction is the re-emission of the radiation by the particles with the same original frequency but in different directions, thus attenuating the radiation in the incident direction. Furthermore, the absorption phenomena take place when, in case of semiconductors inorganic filters (e.g., TiO_2_ and ZnO), the incident radiation have the same energy of band gap. On the contrary, chemical UV filters are organic molecules that use their molecular orbitals to absorb the UV radiation and dissipate the incident light through different mechanism: with radiation emission (such as fluorescence or IR) and without radiation emission. In this last case, the stored energy could be dissipated by molecular vibration, energy transfer to capable molecules, internal conversion (phosphorescence) and, if the energy should be too high, breaking [7]. 

A reported by Suppa and co-workers [8], sunscreen products are essential in skin cancer prevention, but, despite their growing use, the incidence of skin cancer is still increasing. This has been attributed to the wrong application of the products by the users, or even to the excessive concentration of chemical filters necessary to reach the highest protections (SPF 50–50+). Despite the addition of organic filters in sunscreen products leading to filteriing a wide range of UV radiation, thus increasing the SPF, nowadays their use is limited, mainly by the fact that some of them and their degradation products have shown to be persistent and toxic for aquatic environments and responsible for hormone disruptor effects [9,10]. Furthermore, recent studies on skin sensitization has shown that the organic filters butyl methoxydibenzoylmethane, octocrylene, and benzophenone-3 can interact with different skin proteins and initiate processes of skin sensitization [11,12]. Last but not least, some issues related to the safety toward marine environment (i.e., reef) led some countries (Palau, Thailand, Philippines, and Hawaii) to ban some UV organic filters.

The relative inertia of inorganic filters towards skin and other ingredients contained in the formulation, and also their limited penetration on the skin and a wide protection spectrum, led to an increasing use of mineral oxides such as zinc oxide (ZnO) and titanium oxide (TiO_2_) in sunscreen products [3,13]. Despite these advantages, the spread of a micrometric film of mineral oxides particles on the skin tends to give an unwanted whitening effect after its application. This problem has been overcome by reducing at the nanometric level the diameter of the particles. However, the nanomaterial employment in sunscreen products raises remarkable issues regarding their safety towards human health and environment, especially due to their enhanced photocatalytical activity [14,15].

**Figure 1:**
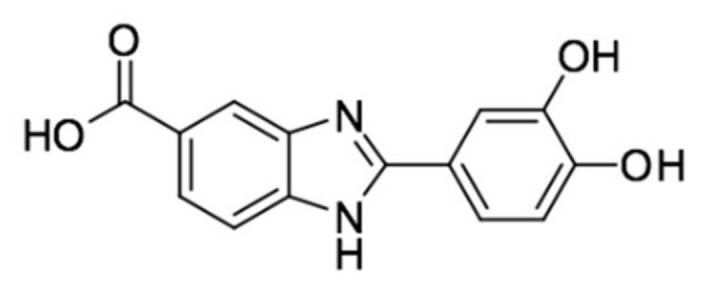
Oxisol structure.

Although the use of nanomaterials in sunscreen formulation, leads to apply a most homogenous film on the skin, with a consequent increasing of SPF value, the adding of specific class of additives called ‘boosters’ in the products, can further enhance the UV protection. Boosters can be small molecules, polymers, or other particles, and mainly act on the rheological properties of the formulation, but also can synergistically interact with the UV filters through antioxidant mechanisms or interfere with the electronic processes of UV radiations absorption. Among these, dihydroxyphenyl benzimidazole carboxylic acid (Oxisol, University of Ferrara European Patent EP2800741) (Figure 1) have shown a strong antioxidant activity linked to its free radical scavenging activity [16]. Oxisol is devoid of significant UV-filtering activity, but endowed with UV-filtering booster capability if associated with known commercial UVB and UVA filters as reported by Bino and co-workers [16]. 

Titanium dioxide (TiO_2_) exists in nature in four natural polymorphs [17]. Rutile is the most stable form and the optical properties of this bi-reflective crystal are its index of refraction in UV and visible ranges. For titanium oxide, the refractive index is around a value of 4.0, for the polycrystalline rutile epitaxially deposited on the skin, and 3.6 for the same anatase film [18]. The TiO_2_ is a semiconductor with an electronic structure characterized by a number of orbital bands separated by a gap energy for which there are no molecular orbitals. Molecular orbitals, very little spaced in terms of energy, are derived from a superposition of a large number of atomic orbitals and form a continuous virtual band. The light radiation with quantum energy equal to the band gap between the valence band (state O-2p for the TiO_2_) and conduction band (Ti-3d state, again for the TiO_2_) allows the passage of the electron e- from the valence band to the conduction band, leaving a hole in the latter (h^+^). Both the e^-^ and h^+^ species can reach the surface of the crystals and take part to redox reactions. For rutile (bulk) the band gap is around 3.03 eV, while for the anatase the value is 3.2 eV8. Due to its semiconductor characteristics, TiO_2_ shows a greater absorption on the UVB, and is one of the few—together with ZnO—inorganic filters listed in the Annex VII of the European regulation, and its use in a micronized form (0.1–10.0 μm) in the cosmetic field has existed for more than 15 years [19], as anatase and rutile, coated and not [20]. However, the mixture of Oxisol and inorganic p-UVf (ZnO and TiO_2_) suffer from some limitations, mainly related to the application’s pH, which is reported to be able to inhibit the antioxidant activity at extreme values, and the increasing of color formulations. As already reported by Nakayama [21], the functionalization of mineral oxide surfaces leads to an increased suspension stability in terms of particle agglomeration, but it can also act on the photocatalytic activity by improving the light absorption mechanisms.

**Figure 2:**
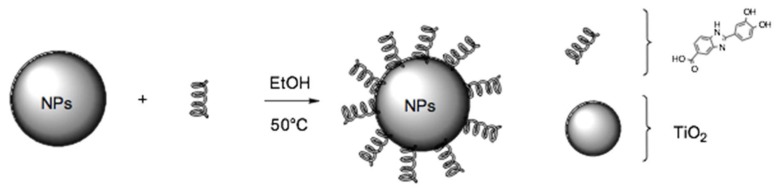
TiO_2_ coating by Oxisol. The reaction was carried out in ethanol at 50 °C for 24 h.

The aim of this work is exploring the performance on UV protection of the new multifunctional ingredient synthetized by means of a direct surface functionalization of an inorganic filter with Oxisol (Figure 2). Moreover, a full characterization of this material has been reported, in order to better understand the data from cytotoxicity tests, photocatalytic activity, and its rheological behavior.

## 2. Materials and Methods 

### 2.1. Materials and Instruments

Titanium dioxide nanometric (Evonik, Evonik Industires AG, Essen, Germany), Titanium dioxide non nanometric (CS Colors srl, Ravenna, Italy), Oxisol (Kalichem, Brescia, Italy), Acid Blue 9 (Framalabor, Milan, Italy), Ethanol BioUltra, for molecular biology, ≥99.8% (absolute alcohol, without additive) (Sigma-Aldrich, Saint Louis, MO, USA), Physiological solution 0.9% NaCl (S.A.L.F. Spa, Bergamo, Italy), UV–Vis spectrophotometer Jasco V-730 (Jasco, Mary’s Court Easton, MD, USA), FT-IR spectrophotometer ATR FT-IR Jasco 4600, ATR PRO ONE (Jasco, Mary’s Court Easton, MD, USA), Centrifuge RE.MI XS R-8D (REMI, Mumbai, India), Turboemulsifier Silverson L5M-A (Silverson, Evry, France), Viscosimeter Brookfield DV2T (Brookfield, Toronto, Canada), Densimeter Mettler Toledo DA-100M (Mettler Toledo, Columbus, OH, USA), Plaster 5 **×** 5 (Leukofix, Hamburg, Germany), Magnetic stirrer (Heidolph, Schwabach, Germany), and WW5 PMMA plates were been purchased (Schonberg GmbH, Munich, Germany). The plates used in this study have an area of 25 cm^2^ and standardized 5 µm roughness, Laboratory glassware Kalsse A. LUMiSizer^®^ (L.U.M.GmbH, Berlin, Germany), Nicomp ZLS Z3000 (PSS, Port Richey, FL, USA). Netzsch 409/C (Gebrüder-Netzsch-Straße 1,995,100 Selb, Germany), Nicomp ZLS Z3000 (8203 Kristel Circle Port Richey, FL, USA).

### 2.2. Titanium Dioxide (TiO_2_) 

**Figure 3:**
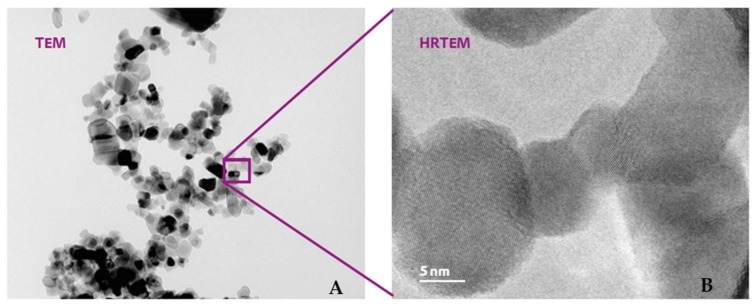
TEM images of nanometric TiO_2_ depicting the primary crystals (**B**) obtained by mean HRTEM and their aggregates and agglomerates (**A**).

TiO_2_ P25 (nanometric form) consists of aggregated primary particles. The aggregates are several hundred nm in size and the primary particles have a mean diameter of approximately 21 nm. Particle size and density of ca. 4 g/cm^3^ lead to a specific surface of approx. 50 m^2^/g. The weight ratio of anatase and rutile is approximately 80/20. Both crystal forms are tetragonal but with different dimensions of the elementary cell. As for non-nanometric titanium dioxide, the dimensions of the primary particles are less than 1.0 micron. More details of titanium dioxide samples used (given by supplier) are shown in Table 1 and Figure 3.

### 2.3. Titanium Dioxide (TiO_2_) Functionalization

This method was used for the functionalization of two types of titanium oxide: nanometric (Anatase: Rutile 3:1, Degussa) and non-nanometric (Anatase: Rutile 1:2). In a 250 mL two-neck flask, 1.0 g of TiO_2_ in 100 mL of ethanol was added; this mixture is placed under continuous stirring and maintained at 50 °C, taking care to wrap the flask in tinfoil in order to avoid photocatalytic reactions. 1.0 g of Oxisol solution in 50 mL of ethanol is added, drop-wise by mean of a funnel, the mixing was continued for 2 h. The reaction was then monitored via UV–Vis spectroscopy, in particular the sample preparation was made withdrawing 5 mL of reaction mixture, centrifuging it for 10 min at 6000 rpm, sampling from the supernatant and filtering through a 0.45-micron filter. 0.10 mL of this solution were brought to volume in a 100 mL flask with ethanol. 3 mL of the solution thus obtained, were placed in a cuvette for spectrophotometric analysis. The analysis was performed at two-hour intervals for the first 3 times, while the final analysis was conducted after 24 h. After this time the reaction has reached completion, the solution was first centrifuged, the liquid phase was wasted and the solid washed twice with distilled water (until perfect transparency and no coloring of the filtered liquid) then dried in thermoblock at 90 °C until constant weight. The dried product is then analyzed by ATR-FTIR spectroscopy.

### 2.4. Evaluation of Stability of Oxisol-TiO_2_ Particles

The test was performed in a 100 mL flask with 0.01g of functionalized product. After taking up to volume with ethanol, ethanol and water mixture (2:3) at pH 2.7, 6.1, and 12.0; and water at pH 2.7, 6.1, and 12.0, the mixture is placed under continuous stirring. The maintenance of the pH was guaranteed by phosphate buffer. The trend of the release is then monitored spectrophotometrically at regular time intervals by withdrawing 0.5 mL solution followed by centrifugation and filtration at 0.45 micron, placing them in a cuvette and diluting to 3 mL with the pure mixture at the pH considered.

### 2.5. Sedimentation Rate

To effectively determine the stability of the dispersed system, a Centrifugal Separation Analysis (CSA) has been conducted by means a multi-sample analytical centrifuge LUMiSizer^®^ (L.U.M. GmbH, Berlin, Germany). This centrifuge is coupled with a spectrometer and employs the STEP™-Technology (Space and Time-resolved Extinction Profiles). The different behavior of the individual samples can be compared and analyzed in detail by tracing the variation in transmission at any part of the sample or by tracing the movement of any phase boundary [22]. By using an algorithm internal to the instrument’s software, the analytical centrifuge can calculate the sedimentation velocity and the hydrodynamic diameter of the suspended phase. The experiments were carried out by loading the different samples (three replicates for four formulation) into the polycarbonate cuvettes at a concentration of 10 mg/L. Measurement was performed with velocity of 2000 rpm at 470 nm laser wavelength; 600 profile number was detected every 10 s.

### 2.6. Photocatalysis

Photocatalytic activity was monitored by the degradation of acid blue 9, after UV radiation, in mixture with pure metal oxide and coated metal oxide [23,24,25]. Dark and light conditions were both investigated in order to remove the dye adsorption contribution. Two mixtures were prepared under the same conditions: 10 mg of photocatalytic material was added to 100 mL of a 0.77 mM solution of Acid Blue 9 in EtOH (0.6 mg in 100 mL). Ethanol was choice in order to better simulate the organic environment when the product will be applied on skin. The first mixture was exposed to UV radiation (320–400 nm) with an 8W lamp at 15 cm distance and room temperature, the second was maintained in dark condition at room temperature as well. This step lasts 1 h, and then the sample is left to stay for 3 h. Before proceeding with UV–Vis analysis (628 nm), the samples were centrifuged and filtered with 0.45 µm. Tests were performed on pure materials (coated TiO_2_) because on formulated products it would requires larger amount of product to achieve significant and reproducible results. In fact, the inhibition of photocatalysis for a pure material (coated and uncoated) can depend only on the presence or absence of the coating, while for the formulation it would depend on multiple factors (i.e., dispersion, homogeneous solubilization, and so on). UVA radiation (320–400 nm) was choice in order to evaluate the protection effects of the new materials on one of the most dangerous and represented radiations in solar light.

### 2.7. Formulations

The study was carried out on different emulsions. Oxisol is present in formulation at a concentration of 0.5% (minimum percentage in order to verify a booster effect), both free and functionalized. Consequently, the amount of TiO_2_ present in the formulation is related to the degree of functionalization of the same, in order to maintain constant the ratio between TiO_2_ and Oxisol in the finished formulation. The formulation compositions are the following: 

Phase A: Steareth-21, steareth-2, glyceryl monostearate, cetearyl alcohol, benzyl alcohol-dehydroacetic acid, coco-caprylate, hihydroxyphenylbenzimidazole carboxylic acid (Oxisol) (when expected);

Phase B: Nanometric TiO_2_ or non-nanometric TiO_2_ or nanometric TiO_2_ functionalized or non-nanometric TiO_2_ functionalized;

Phase C: Panthenol, NaOH, xanthan gum, aqua.

Each formulation was made from the same base in order to minimize the error. The base was made adding the ingredients of phase A except preservative system (benzyl alcohol, dehydroacetic acid) Oxisol (when required) and coco-caprylate, then heating until melt. In a separate beaker, phase C was prepared adding 90% water (heated up to 80–90 °C) and mixing until complete solvation of xanthan gum. When each phase reaches 80 °C temperature, phase C was added to phase A with continuous mixing. The ultimate base was maintained to 70 °C at least.

The formulation procedures were adapted considering the nature of the study products:

a. Formulations without Oxisol: powder of phase B was added to coco-caprylate, then strongly mixed until a homogeneous dispersion was obtained. Under continuous mixing, dispersion was gradually added to base formulation, the mixing goes on until a homogenous emulsion was obtained. Finally, the remaining water was added to system and homogenized.
b. Formulation containing Oxisol alone or as mixture: powder of phase B was mixed with coco-caprylate as reported in point a. In a separate beaker, Oxisol was solubilized in water through quenching with NaOH. Under continuous mixing, the dispersion of powder was added to base formulation, then the Oxisol solution was added too.

The pH and viscosity of each preparation were evaluated (Table 2). The pH of the emulsions is set within a range of 5.5 to 6.5. If required, the pH was corrected with the addition of citric acid. 

### 2.8. Characterization

#### 2.8.1. FT-IR Analysis

The FTIR spectra were collected in the 4000–650 cm^−1^ range, with a resolution of 5 cm^−1^ at room temperature by using a Jasco FT/IR-4600 spectrometer provided with single ATR accessory (Jasco ATR PRO ONE). The number of accumulated scans for each recorded spectrum was automatic whit an average exposure around 1.5 min. All the experiments were performed at room temperature.

#### 2.8.2. TGA and DSC

Thermogravimetric analysis (TGA) and differential scanning calorimetry (DSC) were performed simultaneously by means of Netzsch 409/C instrument. The heating program was set up from 30 °C to 1000 °C, with an increasing of 5 °C min^−1^. Samples (around 15 mg) were placed in a platinum/rhodium crucible and alumina was used for the internal calibration. Measurements were performed in air/N_2_ (40/80 mL/min) mixture.

#### 2.8.3. ζ Potential (DLS-ELS)

The hydrodynamic diameter was measured by means of a multi-angle Nicomp ZLS Z3000 instrument. The dried powders were re-dispersed in ultrapure water (10 mg/L) by probe sonication in an ice bath at 200 W for 10 min (in pulsed 80% mode). All measurements were taken after a pre-equilibration of ca. 5 min. The scattering light was collected with an optical fiber set at 90° scattering angle (W = 25 mW and λ = 639 nm) over at least 6 min at room temperature.

The Zeta potential characterization by electrophoretic light scattering (ELS) were performed by means of Nicomp ZLS Z3000 (PSS, Port Richey, FL, USA). The zeta-potential (Z-pot) values of each sample was determined in the pH 3–11 range and using NaCl as electrolyte (10 mM), following the procedure reported by Brunelli and co-workers [26].

### 2.9. Cytotoxicity

The cytoxicity of TiO_2_ was measured by neutral red assay (NRU) on 3T3 cells. NRU assay was performed in DMEM (Dulbecco’s modified Eagle medium) supplemented with 10% fetal calf serum (FCS), penicillin (100 U/mL), streptomycin (100 μg/mL), and glutamine (2 mM). Cells were seeded in triplicate in 96-well plates at a density of 7 **×** 10^3^ cells/well and treated for 48h with increasing concentrations of the different compounds (1 μg/mL, 10 μg/mL, and 100 μg/mL). Untreated cells were used as a negative control and taken as reference of 100% of cell proliferation.

After treatment, cells were washed and 250 μL of a 25 μg/mL solution of NRU were added. After 2 h, cells were washed again and 150 µL of NRU-desorb solution (49% of water, 50% of 95% Ethanol and 1% of glacial acetic acid) were added. After additional 40 min, the solution absorbance, proportional to the number of live cells, was measured by spectrophotometer at 540nm and converted into % of growth inhibition.

### 2.10. Rheological Measurements of the Emulsions

All prepared emulsions were subjected to rheological measurements using a Brookfield DV2T viscometer. The parameters were set as follows: spindle 25, rotation speed 10 rpm, detection time 30 s.

### 2.11. Oxisol Released from the Emulsions

An exactly weighted amount of emulsion (about 0.30 g) is poured in a 50 mL flask, brought to volume with physiological solution and left under stirring at 37 °C for 4 h. At the end of the 4 h, 10 mL of solution are collected and transferred quantitatively in a centrifuge tube. The centrifugation proceeds for 10 min at 6000 rpm after which filtration is performed. The pure solution is then analyzed by spectrophotometer, withdrawing 0.5 mL and diluting to 3 mL directly in a cuvette with distilled water.

### 2.12. Photochemiluminescence PCL

The PCL assay is based on Popov and Lewin’s method [27] and measures the antioxidant activity of a sample against superoxide anion radicals with a Photochem^®^ apparatus (Analytik Jena, Leipzig, Germany). Radicals are generated from Luminol, a photo-sensitizer agent, as a result of exposure to UV light (Double Bore^®^ phosphorus lamp, output 351 nm, 3 mWatt/cm^2^). The antioxidant capacity was measured using the manufacturer’s ACL (antioxidant capacity of liposoluble substance) kit. The kinetic light emission curve, which exhibits no lag phase in ACL studies, was monitored for 180 s and expressed as micromoles of Trolox^®^ (standard) per gram of dry matter. The areas under the curves were calculated using the PCL soft control and analysis software. The first phase of the analysis consists in the measurement of the blank and in the construction of the calibration curve by means of solutions with a known concentration of the standard (Trolox^®^); the second phase involves the measurement of the antioxidant capacity of the samples. For each sample, the measurements were repeated at least three times. The antioxidant capacity was expressed in micromoles of Trolox^®^ which provide an antioxidant capacity equivalent to one gram of the sample under examination (µmol TE/gram).

### 2.13. In Vitro Evaluation of Filtering Parameters

The in vitro sun protection factor determination method used in this work has been recently proposed by us adapting to UVB the ISO 24443:2012 standard for the in vitro UVA protection determination [28].

In vitro SPF spectrophotometric evaluation was performed measuring absorbance (calculated from transmittance) by a SHIMADZU UV-2600 spectrophotometer provided of integrating sphere ISR 2600 60 mm and coupled with an SPF determination software and a polymethylmethacrylate (PMMA) plate with approximately 15 µL of glycerin served as reference. We used an in vitro approach that involves applying a thin film of product on an artificial substrate that must be as similar as possible to human skin as concerns its physical characteristics. Via spectrophotometric measures, the amount of UV radiation passing through the film can be evaluated. The substrate used for this study was PMMA plates (WW5 PMMA plates have been purchased from Schonberg GmbH, Munich, Germany), a substrate easily handled and that can be supplied with a 5 µm reproducible roughness, with an area of 25 cm^2^ [28]. 

Furthermore, photostability studies were carried out, following the ISO 24443:2012 procedure, with a solar simulator device (Suntest CPS; Atlas, Linsengericht, Germany) equipped with a xenon lamp, an optical filter to cut off wavelengths shorter than 290 nm and an IR-block filter to avoid thermal effects. 

SPF in vitro is calculated as follows from the spectral absorbance characteristics

(1) $$InvitroSPF=\frac{\int_{\lambda =290nm}^{\lambda =400nm}E\left(\lambda \right)I\left(\lambda \right)d\left(\lambda \right)}{\int_{\lambda =290nm}^{\lambda =400nm}E\left(\lambda \right)I\left(\lambda \right)10^{-A\left(\lambda \right)}d\left(\lambda \right)}$$

E (λ), erythema action spectrum (CIE-1987) at a wavelength λ. I (λ), spectral irradiance received from the UV source at a wavelength λ. A (λ), a monochromatic absorbance of the test product layer at a wavelength d (λ), wavelength step (1 nm). 

The UVA protection factor UVAPF0 has been calculated for each non-irradiated plate individually

(2) $$UVAPF0=\frac{\int_{\lambda =320nm}^{\lambda =400nm}P\left(\lambda \right)I\left(\lambda \right)d\left(\lambda \right)}{\int_{\lambda =320nm}^{\lambda =400nm}P\left(\lambda \right)I\left(\lambda \right)10^{-A\left(\lambda \right)C}d\left(\lambda \right)}$$

P (λ) = Persistent Pigment Darkening (PPD) action spectrum. I (λ) = spectral irradiance received from the UV source (UVA 320–400 nm for PPD testing). A (λ) = Mean monochromatic absorbance of the test product layer. C = Coefficient of adjustment. dl = Wavelength step (1 nm). The Critical Lambda describes the amplitude of the protection across all the UV spectra (280–400 nm). More in particular, it is defined as the wavelength at which 90% of the area under the absorbance curve (AUC) is reached starting from 290 nm. The UVA/UVB ratio is defined as the ratio of the mean absorbance from two wavelength ranges (UVA 320–400 nm and UVB 290–320 nm). This value, similarly to the Critical Lambda, provides an evaluation of the amplitude of the protection across the UV spectra without considering the amount of the filtering activity. Values near to 1 are indicative of a broad-spectrum activity.

## 3. Results and Discussion

### 3.1. Adduct Characterization (FT-IR)

**Figure 4:**
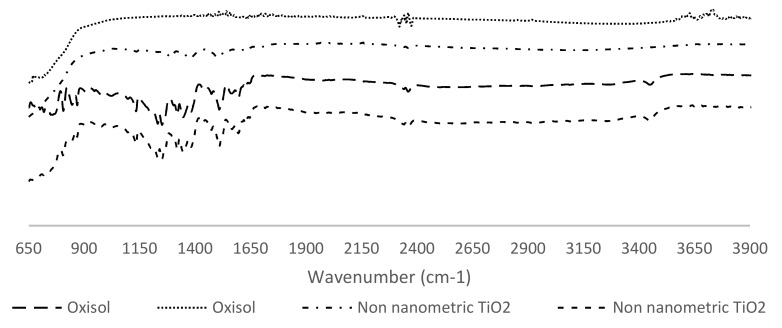
Comparison between FT-IR spectra of TiO_2_, TiO_2_/Oxisol mixture, functionalized TiO_2_, and Oxisol.

TiO_2_ adduct was prepared following the method described in Section 3.1 and the reaction advancement was monitored by UV–Vis spectroscopy. The characterization was performed via ATR-FTIR spectroscopy from 5000 cm^−1^ to 650 cm^−1^ (Figure 4).

**Figure 5:**
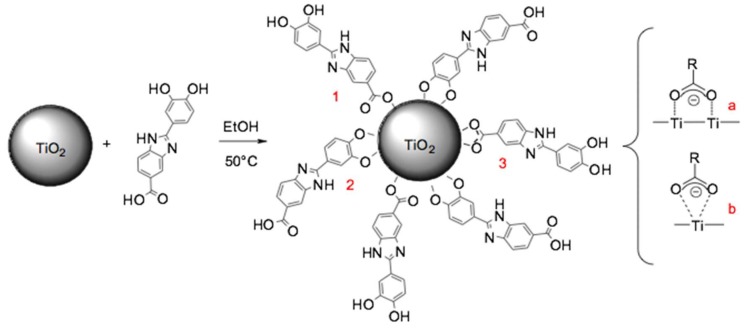
Addition mechanism of Oxisol to TiO_2_ particles. As proved by FT-IR analysis option 1 and 2 are the most expectable (in particular the most frequent would seem option 2), anyway option 3 also can occur via bridge (**a**) or chelating (**b**) addition to titanium.

It was possible to observe the disappearance of signals related to monomeric stretching, ν (COO–H/OH monomer) to 3600/3300 cm^−1^ and that of carboxylic and alcoholic bending, δ (COO–H), 1440–1395 cm^−1^ and δ (OH alcohol), 1420–1330 cm^−1^. In the functionalized form appears the stretching of C=O, ν (C=O) to 1750 cm^−1^. The presence of the carboxyl and the disappearance of the peaks referring to the catechol functions suggests that the functionalization of the titanium dioxide with Oxisol is not chemoselective, but that it can occur through a double path, through both the catechol and the carboxyl functionality. The affinity of the carboxylic and catechol groups to titanium dioxide is reported in literature, in particular, Zeininger et al. [29] carried out a systematic study in order to compare the functionalization of different molecules containing also carboxylic and catechol functionalities from a kinetic and thermodynamic point of view. Wei Lin et al. [30] investigated the a thermodynamic of 3,4-dihydroxybenzoate (CAT) adsorption to zinc oxide colloidal surface. This study has also been taken as a reference to formulate a hypothesis on the coordination of Oxisol. These studies, together with as findings of IR spectroscopy, suggest an effective double way addition of Oxisol to TiO_2_, with a prevalence of catechol addition [29] (Figure 5).

### 3.2. Thermogravimetric Analysis (TGA)

**Figure 6:**
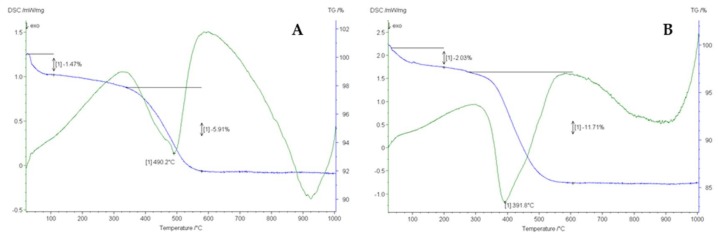
TGA (blue line) and DSC (green line) curves of TiO_2_@Oxisol (**A**) and n-TiO_2_@Oxisol (**B**).

In order to understand the quantity of functionalization, an indirect and direct detection were performed. The indirect detection (described in Section 2.3.) consists on continuous monitoring of Oxisol decreasing in reaction bulk; the presence of plateau corresponds to a saturation of particles site, then a total functionalization. The direct quantification of functionalized Oxisol was detected via TGA analysis in a temperature range between 30–1000 °C. Obtained TGA values are shown in Figure 6 and Table 3.

As regards TiO_2_@Oxisol, the first weight loss corresponding to 1.47% (Figure 6, panel A, blue line) refers to the evaporation of water and therefore to a drying process. The second step corresponding to 5.91% refers to the loss of organic ligand. With reference to the green curve (DSC), on the other hand, there is an endothermic peak at 490.2 °C.

As for nano-TiO_2_@Oxisol, a more important leap can be observed in the TGA curve (Figure 6, panel B, green line) due to a greater functionalization of TiO_2_. The DSC (blue line, panel B) shows a shift at the temperature of 391.8 °C (instead of 490.0 °C as for non-nano TiO_2_). This is probably due to the greater surface area of the system that anticipates the combustion of the organic binder compared to the non-nanometric product.

The functionalization data are in line with what was expected from the coordination of a catechol or a carboxylic acid with TiO_2_, as reported by Zeininger et al. [29].

### 3.3. Colloidal Characterization (CSA, DLS-ELS, ζ)

One of the causes of dispersed systems instability (suspensions, emulsions) is due to complex dynamic behavior of the dispersed phase in solution, which is subjected to agglomeration and sedimentation according to the Stokes’ law. The main effect of these processes is the separation of the phases that compose the formulation.

The measuring of the sedimentation rate is a good index of suspension stability, in fact a lower sedimentation rate means high suspension stability, and consequently great performance of the product during the time. As discussed above, the sedimentation rate also correlates with the hydrodynamic diameter and *ζ* (Z potential) of the dispersed phase. Also, these parameters are essential to understand the relative stability of the formulates. The Table 4 shows the relation between sedimentation rate and particle size detected by DLS (dynamic light scattering) and CSA (centrifugal separation analysis) measurement.

**Figure 7:**
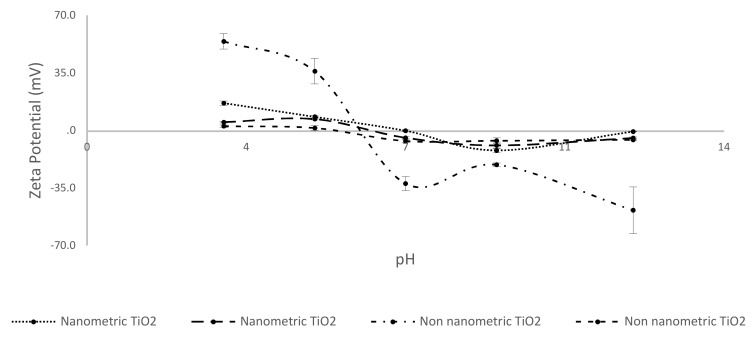
Z potential behavior for each kind of TiO_2_ coated and uncoated.

Particle size extrapolation by CSA and by DLS technique is comparable. Moreover, sedimentation rate shows a better value for coated titanium dioxide. In order to understand if the contribution to the stability was given by steric or electrostatic repulsion, a Z potential analysis was performed (Figure 7). Zeta potential is a parameter linked to the superficial charge density, phenomena of attraction or repulsion in solution, and therefore to the stability of the aggregates. If the value of Zeta potential is high, the electrostatic repulsion prevents the aggregation of the dispersed particles; while when it is low, the attractive forces prevail, and the species give rise to coagulation phenomena.

The value of 30 mV was reached only for functionalized nanometric TiO_2_ that show only a little increase of stability if compared with its uncoated form (Table 4). This evidence suggests that the increased of stability is driven by steric repulsion.

### 3.4. Efficiency Test

#### 3.4.1. SPF

All the tested formulations resulted photostable, as no significant variations in the SPF values have been shown after irradiation conducted, as prescribed by the ISO 24443:2012 procedure, with a solar simulator device (Suntest CPSþ; Atlas, Linsengericht, Germany) (data not shown).

The most interesting results were obtained making the comparison between simple mixture (TiO_2_ + Oxisol in detected percentage) and functionalized TiO_2_ (n-TiO_2_@Oxisol and TiO_2_@Oxisol). The data shows an increasing of SPF in both coated TiO_2_ species (nanometric and non-nanometric) in comparison with TiO_2_ + Oxisol. The increase was higher for nanometric size (with an increase of 73%) and lower for non-nanometric size (19%). Also, the UVA-PF values were better for the functionalization, only UVA/UVB ratio and lambda critical value did not show a better behavior; however, functionalized metal oxide average values are equal for each condition (mixture and functionalization). The functionalization lead to reach a reduction of photon energy needed to transfer an electron into the conduction band, thereby facilitating visible light absorption [31]. Therefore, a decreasing of banh gap could mean, besides an increasing of SPF, a decreasing of photocatalytic effect, with consistent benefits for human health.

#### 3.4.2. Photochemiluminescence PCL

In order to evaluate the influence of functionalization in terms of antioxidant capacity, the emulsions containing the active ingredient as such or in a mixture or functionalized, have been tested by photochemiluminescence (PCL). The PCL analysis was performed with the aim of obtaining information on the antioxidant activity of the TiO_2_@Oxisol complex since the activity is related to the amount of free hydroxyl phenolic groups. 

PCL-based methods differ from other procedures for antioxidant evaluation principally because they do not require oxidizing reagents for the production of the radical species. The most widely used methods for measuring antioxidant activity (Trolox equivalent antioxidant capacity, TEAC I-III; ferric reducing ability of plasma, FRAP; 2,2-diphenylpicrylhydrazyl assay, DPPH; N,N-dimethyl- p-phenyleneamine assay, DMPD) involve the generation of radical species and the presence of antioxidants causing the disappearance of these radicals.

Most of the assays determine the antioxidant activity in the micromolar range, requiring minutes or hours. The photochemiluminescence (PCL) assay presents some advantages: for example, it is more sensitive (nanomolar range) in measuring; in a few minutes, the scavenging activity of antioxidants against the superoxide radical (O_2_•−), which is one of the most dangerous ROS, also occurs in the human body [32] and is responsible for sun-radiation mediated damages. In the PCL assay, the photochemical generation of free radicals is combined with a sensitive detection method using chemiluminescence.

This reaction is induced by optical excitation (hv) of a photosensitiser S which results in the generation of the superoxide radical O_2_•−.

S + h*v* + O_2_ ➔[S*O_2_] ➔ S•+ + O_2_•- (3)

The free radicals are visualized with a chemiluminescence detection reagent, luminol; this acts as photosensitizer as well as oxygen radical detection reagent. The PCL method was carried out as described by Popov and Lewin [33]. 

The results of the test conducted on the antioxidant activity are shown in Table 5. 

Antioxidant power of finished emulsions was detected for mixture and functionalization, for all the materials proposed. The results indicate comparable antioxidant activity, in the respect of the reference Oxisol, for functionalized nanometric TiO_2_ (*p*-value < 0.001) and a consistent lower value for functionalized non-nanometric TiO_2_ (*p*-value < 0.05) and both physical mixtures of nanometric and non-nanometric. The high antioxidant activity, only in the case of the nanometric adduct, could depend from different factors: (i) the higher amount of Oxisol linked by the carboxyl group to nano-TiO_2_ (twice the non-nano form); (ii) the higher surface offered by the nano-form; (iii) the decrease in the TiO_2_ oxidation potential [34] given by functionalization.

### 3.5. Release Test

For all the functionalized particles a stability test was carried out monitoring the ligand release during 4.0 ± 0.5 h in the following condition: pure ethanol, ethanol and water mixture (2:3), and water at pH 2.7, 6.1, 12. These conditions have been chosen to evaluate organic and inorganic environment influence, only organic influence and pH influence. Oxisol release was carried out in order to understand the behavior of adduct both in emulsion and when applied on the skin. The first time, the release from only particles (not in emulsion) was evaluated and the results are shown in Table 6.

For inorganic solvent (water) a higher Oxisol release was observed in alkaline conditions (Table 5). However organic and organic/inorganic environment should be the harshest condition for adduct with the higher release percentage. Nanometric form shows a greater loss (61%) in ethanol and water mixture at pH 12.0. The same trend (greater release in alkaline conditions) was confirmed for non-nanometric form.

Further analyses were performed in order to evaluate the release from emulsions and the results are shown in Table 7.

These data were compared with organic/inorganic environment in Table 6, in fact the emulsion could be assimilated to a mixture of organic and inorganic phases. In accordance with results obtained in Table 7, the values look very similar for the non-nanometric version, which are quite low compared with the nanometric one The percentage decreasing from 9% to 4% in nanometric version could be attributed to a higher stabilization in emulsion, maybe given by the preparation method where functionalized product was dispersed in oil phase then added in water phase. The most efficient dispersion of nano-TiO_2_@Oxisol instead of TiO_2_@Oxisol in oil phase (derivable from Z potential analysis) could explain this phenomenon.

### 3.6. Safety Test

#### 3.6.1. Photocatalysis

As reported by Dimitrovska et al. [28], photocatalytic activity is one of the principal reasons of ROS development driven by inorganic filter. For this reason, we decided to choose titanium dioxide Degussa P25 as the starting material for this study, which has a high photocatalytic activity. In order to understand if the coating lead to a reduction of photocatalytic activity, a test was performed and the results are shown in Table 8.

**Figure 8:**
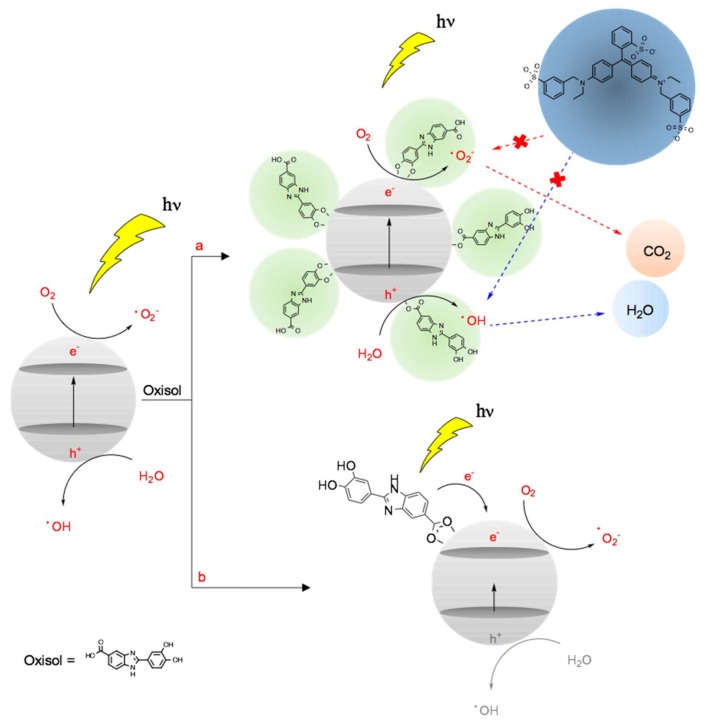
Possible mechanism of photocatalytic quenching. (**a**) Steric hindrance and (**b**) electron injection by ligand: after UV radiation the ligand direct inject electron in conduction band. This significant decrease the hole formation in valence band, then its oxidative activity. Both mechanisms could take place during the tests.

Table 8 shows a little photo-sensitization of dye alone (row 2 and 3) and an efficient decreasing the photocatalytic activity by functionalized TiO_2_. The same experiment carried out in dark condition confirmed that contribute of dye adsorption in particle surface is very low. Furthermore, the decrease in photocatalytic activity in the respect of the free TiO_2_ is not directly related to the functionalized amount but to other mechanisms. In particular, we may advance the hypothesis that the effect might depend on combination of different occurrences: (i) steric hindrance, (ii) direct injection of electron on conduction band by the organic ligand [29]. The radiation in UVA region (320–400 nm) and the related energy is enough to excite the electron in organic molecule but not the electron in valence band (300 nm). By means of functionalization, the oxidative activity, due to bounce from valence band to the conduction band that create an energy hole and a free electron, is reduced limiting the radical generation only to conduction band (Figure 8). The combination of these two phenomena may lead to a reduction of photocatalytic potency. Finally, (iii) Oxisol molecules can act as scavengers for ROS or photogenerated holes/electrons.

#### 3.6.2. Cytotoxicity

Cell growth, in response to increasing concentrations (from 10 to 100 μg/mL) of the different compounds present in the culture media, was assessed on 3T3 cells cultured for 48 h. As shown in Table 9, TiO_2_ compounds did not show cytotoxic effects, even at the highest concentrations used.

## 4. Conclusions

In this study, the functionalization of TiO_2_ with dihydroxyphenyl benzimidazole carboxylic acid (Oxisol) was reported. For TiO_2_, further than our expectations, functionalization resulted in a very effective approach, in particular SPF values of the complexes are better for functionalized metal oxide particles both nanometric and non-nanometric. Furthermore, PCL test show a high antioxidant activity for functionalized nano TiO_2_. The reason could be linked to (i) the higher amount of Oxisol linked by the carboxyl group to nano-TiO_2_ (twice the non-nano form); (ii) the higher surface offered by the nano-form; (iii) the decrease in the TiO_2_ oxidation potential. The Z potential data did not show the expected results with values lower than ±25 mV for each examined adduct. Despite of low value of Z potential, sedimentation rate results considerable reduced these evidences suggest a stabilization via steric contribute. Finally, cytotoxicity shows a substantial inertia of titanium dioxide. We can conclude that coating of TiO_2_ by Oxisol led to several benefit. In particular stabilization in formulation and a booster activity (by means of antioxidant effects), which helps to reduce the amount of inorganic filters to be used in formulation. Photocatalytic activity was significantly lowered and a potent antioxidant activity (comparable to that of the Oxisol itself) was demonstrated by mean of PCL analysis. In our opinion, these results present a new generation of coated inorganic filters, where booster molecule was directly bonded to p-UVf leading to a new material with improved properties, higher stability, and antioxidant potency (this last property is a new feature for inorganic filters).

## 5. Patents

The University of Ferrara has filed the content of the present work: patent appl. no.102019000014076, 8 August 2019.

## Figures and Tables

**Table 1:** Chemical-physical characteristics of the TiO_2_ samples used in the experiments

Properties and Test Methods	Nanometric TiO_2_	Non-nanometric TiO_2_
Specific surface (m^2^/g)	50 ± 20	20 ± 10
pH value in 4% dispersion	4.0 ± 0.5	5.0 ± 0.5
Moisture (wt %) (2 h at 105 °C)	≤1.5	≤0.5
TiO_2_ content (based on ignited material) (wt-%)	>99.5	
Tamped density (g/L)	100–180	90–160

**Table 2:** pH and viscosity data for aqueous TiO_2_ dispersions.

Formulation	pH	Viscosity (ŋ) cP
Only TiO_2_	5.36	20590
TiO_2_ + Oxisol mixture	5.41	15840
Functionalized TiO_2_ (TiO_2_@Oxisol)	5.48	28460
Only TiO_2_ nano	5.30	45700
TiO_2_ nano + Oxisol mixture	6.24	25630
Functionalized TiO_2_ nano (n-TiO_2_@Oxisol)	6.50	34900

**Table 3:** Percentage functionalization of titanium dioxide detected via TGA method. The reported value is the average of at least three measures ± standard deviation (carried out both with the direct and indirect method).

	Weight Loss (%)
**nano-TiO_2_@Oxisol**	10.6 ± 0.6
**TiO_2_@Oxisol**	5.8 ± 0.3

**Table 4:** Particle size values detected via DLS and CSA (first and second columns) and sedimentation rate (last column)

	DLS (nm) ± SD	CSA (nm) ± SD	Sedimentation Rate (µm/s) ± SD
Non nano TiO_2_	343 ± 18	523.9 ± 16	251.3 ± 9
TiO_2_@Oxisol	324 ± 16	328.9 ± 5	80.0 ± 1
Nano TiO_2_	135 ± 7	187.5 ± 3	26.4 ± 2
n-TiO_2_@Oxisol	111 ± 6	166.2 ± 9	20.6 ± 0.2

**Table 5:** PCL results performed on the emulsions containing TiO_2_. Each value was obtained from three different experiments (mean ± SE).

Formulation	µmoli TE/Gram
Emulsion with Oxisol 0.5%	41.96 ± 2.1
Emulsion mixture (Non nan TiO_2_ + Oxisol)	10.37 ± 0.3
Emulsion mixture (Nano TiO_2_ + Oxisol)	1.96 ± 0.02
Emulsion with non nano TiO_2_@Oxisol	11.91 ± 0.1
Emulsion with nano TiO_2_@Oxisol	53.70 ± 3.7

**Table 6:** Oxisol release from particles in different solvents and pH value for coated TiO_2_

Substrate	Solvent	pH	Time (h)	Oxisol Desorption(%)
		12.0		61.43 ± 6.76
	CH_3_CH_2_OH/H_2_O	6.1		9.77 ± 1.95
		2.7		11.05 ± 2.21
Nanometric	CH_3_CH_2_OH	-	4.0 ± 0.5	9.71 ± 2.01
TiO_2_		12.0		1.73 ± 0.31
	H_2_O	6.1		<1.0
		2.7		<1.0
		12.0		7.45 ± 1.86
	CH_3_CH_2_OH/H_2_O	6.1		5.01 ± 1.87
Non		2.7		4.13 ± 1.03
nanometric	CH_3_CH_2_OH	-	4.0 ± 0.5	9.54 ± 0.922
TiO_2_		12.0		7.01 ± 2.01
	H_2_O	6.1		<1.0
		2.7		<1.0

**Table 7:** Oxisol percentage released from emulsions. The percentage are referred to Oxisol linked to TiO_2_.

Substrate	Issue of Release of the Emulsion Adduct	Time (h)	Oxisol Released in the Emulsion test (%)
n-TiO_2_@Oxisol	H_2_O 0.90% NaCl	4.0 ± 0.5	4.36 ± 0.22
TiO_2_@Oxisol	H_2_O 0.90% NaCl	4.0 ± 0.5	4.31 ± 0.25

**Table 8:** Photocatalytic activity of untreated and functionalized TiO_2_. The values are reported as dye concentration: µM and expressed as a percentage (value in brackets) after treatment.

	**Concentration (µM)**	
Only acid blue 9 solution (Dark)	109.99 ± 23.59	
Only acid blue 9 solution (UV)	86.50 ± 18.86	
	**Dye concentration (µM)** **(Nano TiO_2_ form)**	**Dye concentration (µM)** **(Non-nano TiO_2_ form)**
TiO_2_@Oxisol (Dark)	59.64 ± 13.51 (65.60%)	76.31 ± 16.87 (83.93%)
TiO_2_@Oxisol (UV)	47.08 ± 11.03 (51.78%)	57.80 ± 13.14 (63.57%)
TiO_2_ (Dark)	41.74 ± 9.91 (45.91%)	43.68 ± 10.28 (48.04%)
TiO_2_ (UV)	2.70 ± 2.08 (2.97%)	2.18 ± 1.95 (2.40%)

**Table 9:** Cytotoxicity values (cell growth inhibition) obtained evaluating different concentration of powders (1,10,100 µg/mL).

Sample	Concentration µg/mL	% Inhibition ± Standard Deviation
Control	0	0.00 ± 0.00
	1	−2.73 ± 1.78
Nanometric TiO_2_	10	−7.50 ± 1.04
	100	3.08 ± 5.24
	1	−1.37 ± 4.27
Nano-TiO_2_@Oxisol	10	0.18 ± 4.80
	100	4.17 ± 6.20
	1	−1.75 ± 1.77
Non-nanometric TiO_2_	10	−0.84 ± 1.97
	100	0.30 ± 6.02
	1	−4.40 ± 3.03
TiO_2_@Oxisol	10	0.51 ± 8.58
	100	6.34 ± 5.94

## References

[1] Norval M., Cullen A.P., de Gruijl F.R., Longstreth J., Takizawa Y., Lucas R.M., Noonan F.P., van der Leun J.C. (2007). The effects on human health from stratospheric ozone depletion and its interactions with climate change. Photochem. Photobiol. Sci..

[2] Lehmann P. (2011). Sun exposed skin disease. Clin. Dermatol..

[3] Sambandan D.R., Ratner D. (2011). Sunscreens: An overview and update. J. Am. Acad. Dermatol..

[4] Curtis C., Shyr T., Ou-Yang H. (2016). Metal oxide sunscreens protect skin by absorption, not by reflection or scattering. Photodermatol. Photoimmunol. Photomed..

[5] Sayre R., Kollias N., Roberts R., Baqer A. (1990). Physical sunscreens. J. Soc. Cosmet. Chem..

[6] Teixeira M.A.C., Piccirillo C., Tobaldi D.M., Pullar R.C., Labrincha J.A., Ferreira M.O., Castro P.M.L., Pintado M.M.E. (2017). Effect of preparation and processing conditions on UV absorbing properties of hydroxyapatite-Fe2O3 sunscreen. Mater. Sci. Eng. C.

[7] Lautenschlager S., Wulf H.C., Pittelkow M.R. (2007). Photoprotection. Lancet.

[8] Suppa M., Argenziano G., Moscarella E., Hofmann-Wellenhof R., Thomas L., Catricalà C., Gutiérrez-González E., Fargnoli M.C., Peris K., Zalaudek I. (2014). Selective sunscreen application on nevi: Frequency and determinants of a wrong sun-protective behaviour. J. Eur. Acad. Dermatol. Venereol..

[9] Manasfi T., Coulomb B., Ravier S., Boudenne J.L. (2017). Degradation of Organic UV filters in Chlorinated Seawater Swimming Pools: Transformation Pathways and Bromoform Formation. Environ. Sci. Technol..

[10] Rodil R., Moeder M., Altenburger R., Schmitt-Jansen M. (2009). Photostability and phytotoxicity of selected sunscreen agents and their degradation mixtures in water. Anal. Bioanal. Chem..

[11] Stiefel C., Schwack W. (2014). Reactivity of cosmetic UV filters towards skin proteins: Model studies with Boc-lysine, Boc-Gly-Phe-Gly-Lys-OH, BSA and gelatin. Int. J. Cosmet. Sci..

[12] Karlsson I., Persson E., Mårtensson J., Börje A. (2012). Investigation of the sunscreen octocrylene’s interaction with amino acid analogs in the presence of UV radiation. Photochem. Photobiol..

[13] Dransfield G.P. (2000). Inorganic sunscreens. Radiat. Prot. Dosimetry.

[14] SCCS (Scientific Committee on Consumer Safety), Opinion on Tianium Dioxide (nano form), 22 July 2013. http://cosmesispedia.info/wp-content/uploads/2013/06/sccs-TiO2nano.pdf.

[15] Girigoswami K., Viswanathan M., Murugesan R., Girigoswami A. (2015). Studies on polymer-coated zinc oxide nanoparticles: UV-blocking efficacy and in vivo toxicity. Mater. Sci. Eng. C.

[16] Bino A., Baldisserotto A., Scalambra E., Dissette V., Vedaldi D.E., Salvador A., Durini E., Manfredini S., Vertuani S. (2017). Design, synthesis and biological evaluation of novel hydroxy-phenyl-1H-benzimidazoles as radical scavengers and UV-protective agents. J. Enzyme Inhib. Med. Chem..

[17] Kőrösi L., Dömötör D., Beke S., Prato M., Scarpellini A., Meczker K., Schneider G., Kovács T., Kovács Á., Papp S. (2013). Antibacterial Activity of Nanocrystalline TiO_2_(B) on Multiresistant Klebsiella pneumoniae Strains. Sci. Adv. Mater..

[18] Tanemura S., Miao L., Wunderlich W., Tanemura M., Mori Y., Toh S., Kaneko K. (2004). Fabrication and characterization of anatase/rutile–TiO_2_ thin films by magnetron sputtering: A review. Sci. Technol. Adv. Mater..

[19] Gasparro F.P., Mitchnick M., Nash J.F. (1998). A review of sunscreen and efficacy. Photochem. Photobiol..

[20] Smijs T.G., Pavel S. (2011). Titanium dioxide and zinc oxide nanoparticles in sunscreens: Focus on their safety and effectiveness. Nanotechnol. Sci. Appl..

[21] Nakayama N., Hayashi T. (2008). Preparation of TiO2 nanoparticles surface-modified by both carboxylic acid and amine: Dispersibility and stabilization in organic solvents. Colloid. Surf. A-Physicochem. Eng. Asp..

[22] Detloff T., Sobisch T., Lerche D. (2007). Particle size distribution by space or time dependent extinction profiles obtained by analytical centrifugation (concentrated systems). Powder Technol..

[23] Schmidt-Ott A., van den Berg K.J., Dik J., Kooyman P.J., van Driel B.A. (2015). A quick assessment of the photocatalytic activity of TiO_2_ pigments—From lab to conservation studio!. Microchem. J..

[24] Bukallah S.B., Rauf M.A., Ashraf S.S. (2007). Photocatalytic decoloration of Coomassie Brilliant Blue with titanium oxide. Dyes Pigments.

[25] Liu Y., Hua L., Li S. (2010). Photocatalytic degradation of Reactive Brilliant Blue KN-R by TiO2/UV process. Desalination.

[26] Brunelli A., Badetti E., Basei G., Izzo F.C., Hristozov D., MArcomini A. (2018). Effects of organic modifiers on the colloidal stability of TiO2 nanoparticles. A methodological approach for NPs categorization by multivariate statistical analysis. NanoImpact.

[27] Baldisserotto A., Buso P.G., Radice M., Dissette V., Lampronti I., Gambari R., Manfredini S., Vertuani S. (2018). Moringa oleifera leaf extracts as multifunctional ingredients for “natural and organic” sunscreens and photoprotective preparations. Molecules.

[28] Dimitrovska Cvetkovska A., Manfredini S., Ziosi P., Molesini S., Dissette V., Magri I., Scapoli C., Carrieri A., Durini E., Vertuani S. (2017). Factors affecting SPF in vitro measurement and correlation with in vivo results. Int. J. Cosmet. Sci..

[29] Zeininger L., Portilla L., Halik M., Hirsch A. (2016). Quantitative Determination and Comparison of the Surface Binding of Phosphonic Acid, Carboxylic Acid, and Catechol Ligands on TiO2Nanoparticles. Chem. Eur. J..

[30] Lin W., Walter J., Burger A., Maid H., Hirsch A., Peukert W., Segets D. (2015). A general approach to study the thermodynamics of ligand adsorption to colloidal surfaces demonstrated by means of catechols binding to zinc oxide quantum dots. Chem. Mater..

[31] Rangan S., Theisen J.P., Bersch E., Bartynski R.A. (2010). Energy level alignment of catechol molecular orbitals on ZnO(1 1over(2, ¯) 0) and TiO2(1 1 0) surfaces. Appl. Surf. Sci..

[32] Schlester K., Harwat M., Bohm V., Bitsch R. (2002). Assessment of antioxidant activity by using different in vitro Methods. Free Radic. Res..

[33] Popov I., Lewin G. (1999). Antioxidative homeostasis: Characterization by means of chemiluminescent technique. Methods Enzymol..

[34] Higashimoto S., Nishi T., Yasukawa M., Azuma M., Sakata Y., Kobayashi H. (2015). Photocatalysis of titanium dioxide modified by catechol-type interfacial surface complexes (ISC) with different substituted groups. J. Catal..

